# Reviewer-coerced citation: case report, update on journal policy and suggestions for future prevention

**DOI:** 10.1093/bioinformatics/btz071

**Published:** 2019-01-30

**Authors:** Jonathan D Wren, Alfonso Valencia, Janet Kelso

**Affiliations:** 1 Arthritis and Clinical Immunology Research Program, Division of Genomics and Data Sciences, Oklahoma Medical Research Foundation, Oklahoma City, OK, USA; 2 ICREA, Barcelona, 08010, Spain & Barcelona Supercomputing Center (BSC), Barcelona, Spain; 3 Department of Evolutionary Genetics, Max Planck Institute for Evolutionary Anthropology, Leipzig, Germany

A case was recently brought to the journal’s attention regarding a reviewer who had requested a large number of citations to their own papers as part of their review. After investigation of their most recent reviews, we found that in every review this reviewer requested an average of 35 citations be added, ∼90% of which were to their own papers and the remainder to papers that both cited them extensively and mentioned them by name in the title. The reviewer’s phrasing strongly suggested that inclusion of these citations would influence their recommendation to the editor to accept or reject the paper. The reviewer was unable to provide a satisfactory justification for these requests and *Bioinformatics* has therefore banned them as a reviewer. Our investigation also suggests that the reviewer has behaved similarly in reviewing for other journals. This case has alerted us to how the peer-review system is vulnerable to unethical behavior, and prompted us to clarify the journal’s policy on when it is appropriate for reviewers to request citations to their own work, and to suggest how some of the current weak points in the peer-review system can be mitigated, so that this behavior can be detected more quickly and efficiently.

## 1 Peer-review is the core of the editorial process and the basis of the publication system

Peer-reviewers are typically selected based on their expertise in the areas of research associated with newly submitted manuscripts. They are among the most likely to be familiar with prior publications pertinent to the submission, and reviewer feedback on the completeness and accuracy of a manuscript’s reference list is desirable and welcome. It is therefore not unusual that one or a few requested citations may be to the reviewer’s own research. However, reviewers should be aware that a rigorous scientific justification for the inclusion of a new citation must be provided. Since it is easy to provide a tenuous justification for inclusion, as this reviewer often did (e.g. that his papers also involved analysis of sequence data), it should instead be stated why the authors would be remiss or the paper weaker if the citation were not included. Likewise, editors and authors should be aware of the imbalance of power that exists in the review process, and should ensure that any citations added in response to a reviewer comment are relevant and important.

## 2 The nature of the problem for science in general

Citations have been called the ‘currency’ of science, meaning that they could be considered a quantifiable and objective metric of the impact of a scientist’s research. Specific metrics, such as the H-index, are intended to reflect this and scientists therefore have an incentive to try to improve their H-index. Indeed, the reviewer we caught requesting extensive citation of their work has a webpage that includes prominent mentions of both their high H-index and past awards they received from Thomson Reuters for being a highly cited researcher. When a reviewer agrees to review a paper with the intention of inflating the number of citations to their work, this is a conflict of interest and an unethical manipulation of the peer-review system.

One might ask how this reviewer got away with submitting multiple reviews containing coercive requests for citation before being banned. The shortest explanation is that excessive self-citation demands are generally not seen as an ethical problem *until* a pattern is established, and a decentralized peer-review system is not amenable to detecting patterns. Editors may overlook requests, authors seem reluctant to bring it up explicitly to the editor, reviewer comments are anonymous and scattered across different editors and different journals, and even editors that spot such patterns may not even be aware what options they have, and therefore take the least-energy option of no longer inviting that reviewer. Because accusing someone of unethical conduct is a serious matter, the editors and authors involved are hesitant to do so, particularly if all they have is one instance to base their actions upon.

Combined, this creates a system whereby such behavior can persist for a very long time. Even in the rare event that such misbehavior is detected, there is no global solution. Following the guidelines of the Committee on Publication Ethics (COPE), the first step is to contact the reviewer for an explanation. If it is unsatisfactory, then bring the matter to the attention of their immediate supervisor. This second step is only effective in a well-organized academic system, which was not the case here. Despite the actions taken by *Bioinformatics*, it is likely that this reviewer will continue to review for other journals after this editorial is published. Readers have no doubt noticed that this reviewer is not mentioned by name. This is because we have concluded that we have an obligation to maintain peer-reviewer anonymity, and thus, we can only alert others to the general problem. We debated this last point extensively, and have raised the issue with the COPE as a case report to be discussed.

Specific suggestions for reviewers:

1. **Motivation**. Reviewers should properly motivate their requests for citations and specify how strongly they feel about the addition of references. For example, saying ‘*the authors’ review of the field is incomplete, they should add the following references*’ is vague, whereas ‘*similar studies on the use of X for the purpose of Y were published prior to this one and are needed to alert readers to prior art*’ is specific. The less specific a reviewer is regarding motivation, the less weight their request should be given.
2. **Moderation**. Reviewers should refrain from requesting substantial numbers of references. What is ‘substantial’ will vary by the type of article, with review articles expected to be better in their coverage and short two-page application notes expected to include only the most relevant references. We propose a general rule of thumb to define ‘substantial’ as requesting addition of more than one reference per printed journal page of the paper. In the event the authors’ citations of pertinent prior research is highly incomplete, a reviewer should simply say so and then point them in the right direction with a few citations and let them do the rest.
3. **Communication**. If a reviewer notices another reviewer has requested excessive or unmerited citations and this has not been commented on by the editor, they should feel free to share their observations and opinions directly with the editor. Reviewers should be cognizant they are also in a position to recognize patterns of abuse from their fellow reviewers.

Specific suggestions for journals:

1. **Document patterns**. Manuscript handling systems should include a checkbox for each reviewer that asks ‘did this reviewer request citation to their own research?’ Editors with a concern about reviewer’s citation requests could then see what percentage of reviews returned contained self-citation requests.
2. **Brief but clear guidelines.** Instructions should be kept simple and clear. As a result of this case we have updated our reviewer guidelines to state that requests for citations should include ‘*a brief, yet specific, rationale as to why their inclusion is merited. This rationale is particularly important if the reviewer requests citation of their own papers*.’

Specific suggestions for authors:

1. **Voice concerns**. Although adding multiple references in response to a reviewer request might seem like an ‘easy’ way to satisfy at least one of the reviewers, each unmerited citation clutters your paper and rewards unethical behavior. Don’t be hesitant to include in your response that you have considered the suggestion and feel they are not necessary. In the event the reviewer responds negatively, you should contact the editor for guidance. This is in accordance with the Ethical Guidelines for Reviewers published by the Committee on Publication Ethics (COPE Council. *Ethical guidelines for peer reviewers*. September 2017. www.publicationethics.org) Specific suggestions for editors handling papers:
2. **Vigilance**. The ultimate responsibility in preventing this behavior lies with the editors handling the papers. Careful consideration of the referee reports to detect unethical behavior, including unjustified requests for citations, particularly citations of the reviewer’s own work, is important and all efforts should be made to prevent such requests being made to the authors. Importantly, when a reviewer requests substantial self-citation, this should be reported to the journal so they can investigate whether or not this is part of a pattern, as in the specific case discussed here. *Bioinformatics* acknowledges that their editorial controls have failed for some time in this particular case, and sincerely apologizes to our authors, referees and readers for not detecting this sooner.

## 3 Conclusions

This phenomenon of reviewer-coerced citations is not new (Huggett, 2013; Ioannidis, 2015; Resnik *et al.*, 2008; Thombs and Razykov, 2012; Thombs *et al.*, 2015; Wilhite and Fong, 2012), but also not very well explored in terms of how extensive it may be or how it should be dealt with. We hope this editorial will prompt some discussion on the appropriate balance between the need for peer-reviewer anonymity and the need to alert others to potentially unethical behavior once a pattern is established, particularly when it is difficult to detect such patterns. It is possible that eliminating some of the anonymity, either by open peer-review or publishing anonymized peer reviews alongside accepted papers may disincentivize this behavior. Similarly, because highly centralized research resources, such as Publons and ORCID, have been developed, we hope that some ideas or discussion could take place regarding how these or similar centralized resources could be used, responsibly, to help document patterns of ethical concern that are otherwise difficult to detect. 

*Conflict of Interest*: none declared.
